# An Animal Model of Liuzijue Based on Kinematic Features Exploration: A Pilot Study Conducting in COPD

**DOI:** 10.1002/iid3.70233

**Published:** 2025-08-13

**Authors:** Jian Li, Xiang Ji, Kangxia Li, Min Cao, Yuxin Sun, Chengbing Cao, Zifei Yin, Xin Wang, Fanfu Fang, Cai‐tao Chen, Wei Gu

**Affiliations:** ^1^ Faculty of Traditional Chinese Medicine Naval Medical University (Second Military Medical University) Shanghai China; ^2^ Department of Sports Rehabilitation Shanghai University of Sport Shanghai China; ^3^ Department of Rehabilitation Medicine First Affiliated Hospital of Naval Medical University, Naval Medical University (Second Military Medical University) Shanghai China; ^4^ Department of Rehabilitation Medicine, Shanghai Fourth People's Hospital, School of Medicine Tongji University Shanghai China

**Keywords:** animal model, chronic obstructive pulmonary disease, kinematic feature, Liuzijue, pulmonary rehabilitation

## Abstract

**Background:**

Liuzijue is an essential nonpharmacological intervention within the comprehensive management strategies for chronic obstructive pulmonary disease (COPD) patients. However, the absence of an animal model for Liuzijue presents methodological limitations in its basic research. This study aimed to apply interventions with kinematic characteristics of Liuzijue to COPD mice, with the objective to explore an animal model of Liuzijue suitable for experimental studies.

**Methods:**

Forty‐eight C57BL/6 mice randomly assigned into six groups to receive interventions mimicking Liuzijue's kinematic features, namely aerobic exercise, pursed‐lip breathing and abdominal breathing. Post‐intervention, respiratory function, diaphragmatic contractility, and rectus abdominis thickness were assessed. Histological structures of lung tissue, diaphragm, and rectus abdominis were observed using H&E staining. Expression levels of IL‐10, INF‐γ, and TNF‐α in bronchoalveolar lavage fluid, and p65, CasP3, MuRF1, MyoD1, IGF‐1, and Hspa5 in the diaphragm and rectus abdominis were measured.

**Results:**

The modeling process impaired respiratory function and diaphragmatic contractility in mice. All four stimulation forms effectively improved pulmonary and diaphragmatic function in COPD mice. The thickness of the rectus abdominis was increased by three specified exercise forms. Despite minimal lung tissue structural changes, swimming and abdominal stimulation improved the structure of the rectus abdominis in COPD mice and airway inflammation levels were inhibited. Lastly, the four stimulations regulated the balance of myoprotein synthesis and degradation in the diaphragm and rectus abdominis, although the intervention effects of the four stimulations did not escalate with the complexity of the methods.

**Conclusion:**

The exercise stimulation paradigms established by simulating the kinematic characteristics of Liuzijue possess the therapeutic effects of Liuzijue in improving respiratory function, demonstrating first evidence that kinematic‐based animal models can bridge traditional Qigong and modern mechanism research. However, the development of an animal model for the application of Liuzijue in basic research still warrants further exploration.

## Graphical abstract

In this study, we initiated an exploration based on the kinematic characteristics of Liuzijue Qigong, aiming to develop an animal model of Liuzijue suitable for animal experimentation. The outcomes of the research have substantiated the feasibility of this model establishment protocol. However, we acknowledge that there is scope for further optimization of this model to enhance its applicability and reliability in future studies.

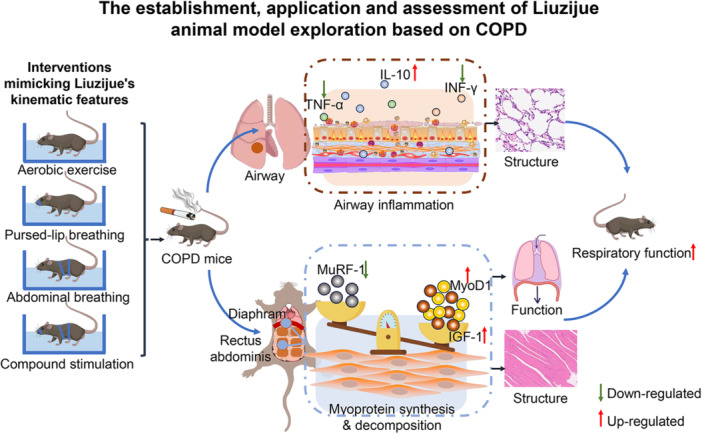

## 1 Introduction

Chronic obstructive pulmonary disease (COPD) has emerged as a significant respiratory condition impacting global health, given the prevalence of chronic noncommunicable diseases in global mortality [1]. By 2016, COPD had become the third leading cause of death globally, resulting in over 3 million deaths annually, a trend projected to continue until 2030 [2]. The disease poses a major health challenge in the upcoming decades, driven by ongoing exposure to tobacco smoke and environmental pollutants, an aging population, and the absence of a cure. Notably, beyond the direct lung damage, COPD patients experience various extrapulmonary effects due to chronic systemic inflammation [3], affecting respiratory muscles, skeletal muscles, brain, heart, and other organs, manifesting at any disease stage. Controlling systemic inflammation and regulating immunity are the main strategies for COPD treatment [4, 5], and current drug treatments have been shown to improve respiratory symptoms, quality of life, and reduce the frequency of exacerbations in patients with COPD. However, even with optimized drug combinations and innovative inhalers, multiple patients continue to experience high levels of symptoms, often with unresolved comorbidities. This situation has prompted researchers to redirect their attention towards nondrug interventions for COPD and new pathways to control systemic inflammation. The Lancet Commission recently advocated for a reevaluation of COPD treatment [6], emphasizing the need for more integrated management approaches.

For a considerable period, the comprehensive intervention mode represented by traditional Chinese medicine has played a pivotal role in managing COPD, forming an integral part of the disease management for numerous COPD patients, particularly through Chinese Qigong such as Liuzijue [7]. Liuzijue is a Taoism‐originated regimen combining the Tuna (breathing in and out) and moderate exercise training. It was first published in Tao Hongjing's “The Yang Xing Yan Ming Lu” during the Northern and Southern Dynasties, and was re‐formulated by the General Administration of Sport of China in 2007 to form a complete set of standard Qigong training methods, including the pronunciation and exhalation of the six characters “xu,” “he,” “hu,” “si,” “chui,” and “xi.” Liuzijue covers the rehabilitation elements of modern pulmonary rehabilitation, such as exercise training, respiratory muscle training and psychological rehabilitation, and meets the requirements of pulmonary rehabilitation in most cases. Clinical studies have demonstrated the positive impact of Liuzijue on COPD patients. Following intervention, patients with acute exacerbation of COPD showed significant improvements in pulmonary function, exercise capacity, and quality of life [8]. Our previous research also supported the rehabilitation benefits of Liuzijue in enhancing respiratory status and exercise capacity in patients in remission period of COPD [9, 10]. However, the current understanding of the underlying mechanisms behind the rehabilitative effects of Liuzijue remains rooted in traditional Chinese medicine theory. The specific molecular and biological mechanisms of this exercise are still in their early stages of exploration, particularly in the delayed investigation and validation of animal models for Liuzijue. Therefore, it is imperative to allocate adequate attention to the exploration and validation of Liuzijue animal model suitable for basic research.

Modern rehabilitation theory holds that Liuzijue is a comprehensive recreational exercise that engages the entire body and possesses unique characteristics [11]. (1) Liuzijue involves pursing the lips and increasing exhalation resistance through the pronunciation of the six words “xu,” “he,” “hu,” “si,” “chui,” and “xi,” akin to pursed‐lip breathing in contemporary rehabilitation training. (2) Liuzijue incorporates deep and slow breathing as well as reverse abdominal breathing to target core respiratory muscles, such as the abdominal and diaphragmatic muscles. (3) The physical movements within Liuzijue provide a holistic workout for the entire body, constituting a low‐intensity aerobic exercise. The movement characteristics observed throughout the Liuzijue process represent the initial findings in understanding the rehabilitation effects of Liuzijue through modern rehabilitation theory. While these findings do not fully uncover the specific mechanisms of Liuzijue, they do offer a valuable research pathway for future studies. Additionally, when compared to Taichi and other Qigong practices, Liuzijue features simpler breathing movements and clearer stimulation of target organs, making it feasible to simulate Liuzijue's movement characteristics.

In this study, we replicated the COPD animal model through tobacco smoke exposure and conducted interventions on the modeled mice. The intervention process emulated the principal kinematic features of the traditional Chinese Qigong Liuzijue: augmenting the respiratory load in the perioral region, activating the core respiratory muscle groups, and integrating low‐intensity aerobic activities. We further dissected the roles and underlying mechanisms of these kinematic attributes in the rehabilitative efficacy of Liuzijue, focusing on the impact of interventions mimicking the kinematic features of Liuzijue on both pulmonary and extrapulmonary inflammation levels in COPD, and exploring the potential of this intervention to modulate immunity, given the crucial role of inflammation and immune modulation in the pathogenesis [4, 5, 12] and treatment [13, 14, 15] of COPD. Therefore, this study aimed to establish and validate a novel animal model replicating the core kinematic features of Liuzijue (pursed‐lip breathing, abdominal muscle engagement, and low‐intensity aerobic exercise) in COPD mice. Utilizing this model, we sought to investigate the therapeutic effects of these simulated Liuzijue features on respiratory function and to dissect their underlying mechanisms, with a particular focus on their impact on pulmonary and systemic inflammation, as well as their potential to modulate immune‐related pathways, which are central to COPD pathogenesis and treatment [4, 5, 12, 13, 14, 15]. We anticipate that this foundational model will provide a valuable tool for elucidating the mechanisms of Liuzijue and serve as a selectable reference for future basic research on traditional Chinese nonpharmacological interventions for COPD.

## 2 Methods

### 2.1 Animals

**Figure 1:**
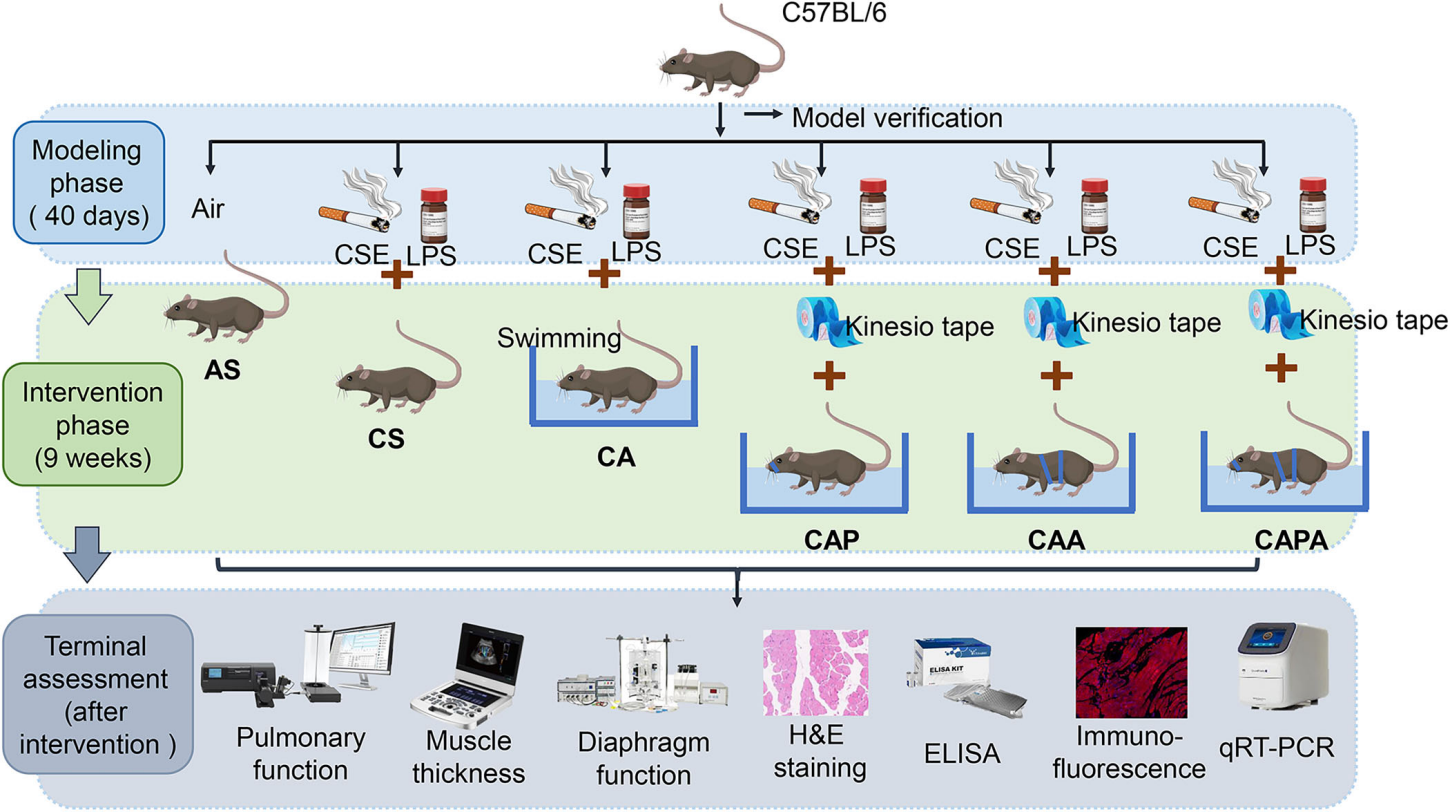
Schematic diagram of the establishment, application and assessment plan of Liuzijue animal model exploration based on COPD. Mice were randomly assigned into six groups: AS, CS, CA, CAP, CAA, and CAPA (*n* = 8). Except for the AS group, which served as the control, all other groups were subjected to a 40‐day regimen of cigarette smoke exposure and intra‐airway LPS instillation to establish the COPD mouse model. Subsequently, the CA, CAP, CAA, and CAPA groups underwent a 9‐week intervention involving various forms of exercise. Ultimately, at the end of the 9‐week intervention period, mice were subjected to the following assessments in sequence: pulmonary function testing and rectus abdominis thickness measurements under anesthesia; BALF sampling, tissue collection for diaphragmatic contractility, histopathology, and molecular analyses. AS, blank group; CA, aerobic exercise group; CAA, abdominal muscle stimulation group; CAP, pursed lip breathing group; CAPA, compound stimulation group; CS, model group; CSE, cigarette smoke exposure; ELISA, enzyme‐linked immunosorbent assay; H&E staining, hematoxylin and eosin staining; LPS, lipopolysaccharide; qRT‐PCR, quantitative reverse transcription polymerase chain reaction.

Male 8‐week‐old C57BL/6 J mice, obtained from Shanghai Jiesjie Laboratory Animal Co., LTD., were housed in a controlled environment with a 12:12‐h light‐dark cycle, and had ad libitum access to food and water. The mice were randomly assigned to six groups using random numbers (eight mice per group, see Figure 1): blank group (sedentary + air, AS), model group (sedentary + cigarette smoke exposure, CS), aerobic exercise group (cigarette smoke exposure + aerobic exercise, CA), pursed‐lip breathing group (cigarette smoke exposure + aerobic exercise + pursed‐lip breathing, CAP), abdominal muscle stimulation group (cigarette smoke exposure + aerobic exercise + abdominal stimulation, CAA), and compound stimulation group (cigarette smoke exposure + aerobic exercise + pursed‐lip breathing + abdominal stimulation, CAPA). All animal groups were housed under identical conditions. The research protocol was approved by the Science and Technology Ethics Committee of Tongji University (No. TJBH07824101). All procedures adhered to the 3 R principle and had predefined humane endpoints: > 20% body weight loss within 48 h, labored respiration unresponsive to oxygen, or self‐isolation/inability to access food/water. No animals met these endpoints during the study.

### 2.2 Cigarette Smoke Exposure

During the initial 40‐day modeling phase, the smoke exposure plan was modified based on our previous research [16, 17, 18]. Briefly, 20 commercially filtered cigarettes (11 mg tar, 0.8 mg nicotine, and 13 mg carbon monoxide per cigarette) were ignited in two separate sessions and passed through a passive smoke exposure system (PAB‐S200; Beijing Bestlab High‐Tech Co. Ltd. Beijing, China) to generate cigarette smoke. The mice were exposed to the smoke for 1 h twice a day, 6 days a week, for 4 weeks. During the smoke exposure process, the carbon monoxide concentration in the chamber was maintained at 310‐380 ppm, with the oxygen concentration not falling below 18%. The model mice also received lipopolysaccharide (LPS, L2630, Sigma‐Aldrich, Darmstadt, Germany) (750 ng/kg dissolved in 50 μL normal saline) intranasally under anesthesia (2% pentobarbital sodium, 80 mg/kg) on Day 1 and day 14, followed by placement in a cage after rotating on a turntable for 10 s. AS mice received ambient air via the passive exposure system for 1 h twice daily without cigarette smoke and underwent sham intranasal instillation (50 μL saline without LPS) on Day 1 and Day 14 under anesthesia, followed by turntable rotation. Notably, CS mice controls for COPD pathology development without therapeutic confounders.

### 2.3 Exercise Protocol

Following the establishment of the COPD model, all mice in four exercise groups underwent adaptive swimming training for 1 week. The mice were placed in a water tank for the intervention, with the water level initially set at 10 cm and adjusted accordingly to the mice's body length. The water temperature was maintained at 33°C‐35°C. The aerobic intervention process was divided into two stages: the first week served as the adaptive intervention stage, where mice swam for 10 min on the first day, increasing by 10 min daily until reaching 30 min by the third day, with training lasting for 6 days. From the second week onwards, mice underwent weightless swimming training once daily, 5 days a week, with each session lasting 30 min. To prevent mice from floating on the water's surface, experimenters used a stick to stimulate the mice's tail and encourage movement. Additionally, tired mice were promptly removed from the water for a 2‐min rest. Strict sedentary status maintained in both AS and CS groups in same environment.

### 2.4 Pursed‐Lip Breathing

**Figure 2:**
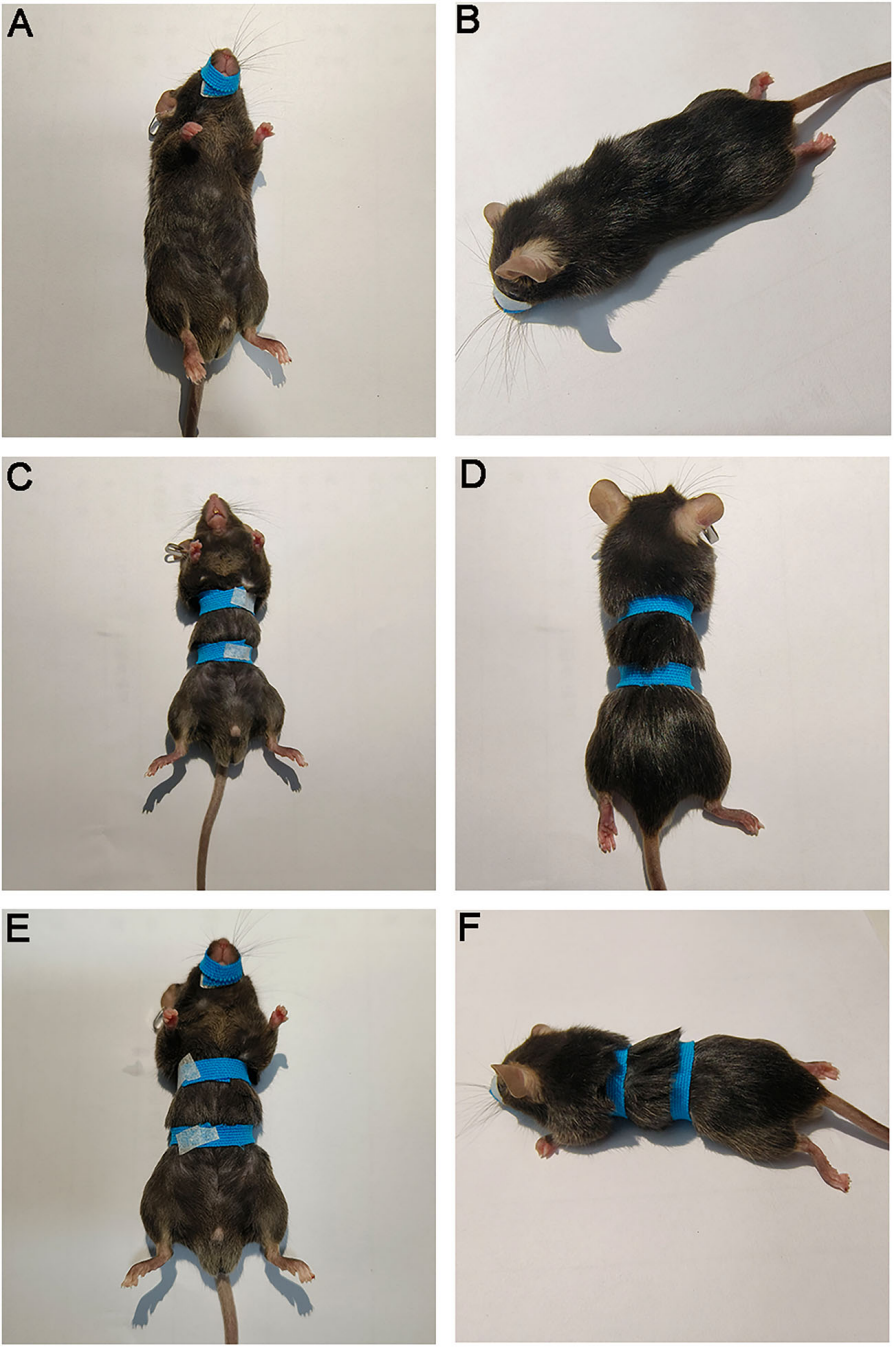
Depiction of kinesiology taping mimicking the kinematic characteristics of Liuzijue Qigong. Panels illustrate the application of kinesiology tape on mice in the CAP group in ventral (A) and dorsal (B) views; the CAA group in ventral (C) and dorsal (D) views; and the CAPA group in ventral (E) and dorsal (F) views. The taping technique is designed to replicate the kinematic features of Liuzijue Qigong, targeting specific muscular engagement and respiratory patterns. AS, blank group; CS, model group; CA, aerobic exercise group; CAP, pursed lip breathing group; CAA, abdominal muscle stimulation group; CAPA, compound stimulation group.

In the current study, the muscular periphery of the mice's lips was strategically engaged through the application of kinesio tape to modulate oral aperture and promote specific respiratory efforts (see Figure 2). The kinesio tape was tailored into 3 × 50 mm I‐straps, utilizing the mouse's chin as the foundational anchor point. To standardize the degree of lip pursing, a tension meter (Tensometric, Germany) was used to apply 30% tension along the longitudinal axis. The tape was extended from the chin to the nasal bridge, resulting in a 130% increase in tape length relative to the resting state. The trailing end was affixed at the nasal bridge. Lip closure efficacy was verified by visual confirmation of reduced oral aperture (< 1 mm) and absence of nasal flaring during respiration. The extremities of the tape were secured with paper tape, maintaining uniform tension while meticulously avoiding contact with the mouse's whiskers and ocular region (see Figure 2A,B). Subsequent to the tape application, the orifice between the lips was inspected to ensure proper closure. The mice then underwent a swimming exercise protocol in a water tank, simulating a weightless state for a duration of 30 min, executed five times per week. This aquatic regimen was designed to correspond with the exercise paradigm of the CA group, thereby allowing for a controlled assessment of the intervention's impact on respiratory mechanics and physical conditioning.

### 2.5 Abdominal Muscle Stimulation Training

The mice's chest and abdominal muscles were taped with kinesio tape, applied with varying degrees of tension to facilitate specific respiratory patterns. The kinesio tape was cut into 3 × 50 mm I‐strap to fix and support the ribcage and abdomen of the mice. For thoracic immobilization, the tape was anchored at the mouse's right axilla, and a calibrated tension meter applied 50% tension across the transverse axis. The tape was then encircled around the thoracic cavity, achieving a 150% length extension from baseline. Thoracic restriction was confirmed by observing dominant abdominal wall movement during breathing cycles. As for abdominal support, the right lateral abdomen served as the anchor point. A 30% tension was applied using the tension meter along a 0.5 cm transverse line superior to the iliac crest, extending the tape to 130% of its original dimension. Resistance to abdominal expansion was validated by palpating increased abdominal muscle contraction during inspiration. The tape's end was affixed on the right abdominal wall, with final securement accomplished using paper tape. It was essential to apply consistent tension and ensure proper alignment with the 1 cm transverse line above the iliac crest's highest point during the wrapping procedure. The abdominal support kinesio tape provided resistance to abdominal breathing in mice, targeting the muscles involved in abdominal respiration (see Figure 2C,D). Postapplication of the tape, the mice participated in unencumbered swimming sessions within a water tank for 30 min, conducted five times weekly, adhering to a protocol analogous to that of the CA group.

### 2.6 Compound Stimulation

The mice in CAPA were subjected to a specialized training regimen incorporating pursed‐lip breathing and abdominal respiration exercises, facilitated by the strategic application of kinesio tape. After the nasolabial, thoracic, and abdominal muscles were secured and supported using kinesio taping techniques (as illustrated in Figure 2E,F), the mice were introduced to an aquatic environment within a water tank. They underwent a swimming intervention, devoid of additional load, for a duration of 30 min, executed five times weekly. The procedural guidelines for applying the kinesio tape adhered to the established protocols utilized for the CAP and CAA groups. The swimming exercise program was designed to replicate the established regimen of the CA group, thereby ensuring a standardized approach to evaluating the impact of the intervention on the physiological outcomes of interest.

### 2.7 Adverse Event Monitoring

All sessions were directly supervised by trained researchers, who assessed the mice every 10 min for respiratory distress (gasping, cyanosis), impaired mobility (inability to maintain swimming posture), and signs of exhaustion (prolonged flotation despite stimulation). Any mouse meeting these criteria was immediately removed for a 15‐min rest with supplemental oxygen, and training was terminated if symptoms persisted. Thanks to the graded exercise protocol, no adverse events requiring termination occurred.

### 2.8 Terminal Assessment

At the end of the 9‐week intervention period, mice were subjected to the following assessments in sequence: pulmonary function testing and rectus abdominis thickness measurements under anesthesia; bronchoalveolar lavage fluid (BALF) sampling, tissue collection for diaphragmatic contractility, histopathology, and molecular analyses.

### 2.9 Pulmonary Function

Mice were anesthetized with 2% pentobarbital sodium (0.25 mL/100 g) via intraperitoneal injection. Following aseptic preparation, the cervical region of the mice was incised longitudinally. Subcutaneous tissue and cervical musculature were gently dissected to isolate the trachea, which was then exposed and mobilized. A horizontal incision was made through the tracheal rings using fine tissue scissors, facilitating the insertion of a tracheal cannula. Respiratory system compliance (Crs), quasi‐static compliance (Cst), respiratory system elasticity (Ers), inspiratory capacity (IC), airway resistance (Rn) and respiratory resistance (Rrs) were measured through the flexiVent small animal ventilator (SCIREQ, Shanghai, China).

### 2.10 Diaphragmatic Contractile Function

Tissue perfusion system (ADInstruments, Michigan, AUS) and data acquisition and processing equipment (Power Lab 4/35, ADInstruments, Michigan, AUS) were used for in vitro muscle strength testing [19]. Krebs‐Hanseleit buffer was prepared at 37°C and the gas mixture of 95% O_2_–5% CO_2_ was premixed for 15 min. A 2 g weight load calibration was performed. The diaphragmatic muscles of mice were dissected under deep anesthesia following chest opening. Several 2.5 mm strips were cut along the central tendon and placed in Krebs‐Hanseleit buffer at 37°C. One end of the muscle strip was secured at the bottom of the bath, while the other end was attached to the tension sensor. After a 10‐min balance period, electrical stimulation was applied to induce contraction of the diaphragm muscle strip, and the contraction degree was recorded.

### 2.11 Rectus Abdominis Thickness

The thickness of the rectus abdominis muscle in mice was measured using the Acclarix AX2 Exp (EDAN, Shenzhen, China) portable musculoskeletal ultrasonic diagnostic instrument. After ensuring the stabilization of the mice's respiratory patterns, the rectus abdominis muscle was scanned using two‐dimensional gray scale mode. The detection area of rectus abdominis was fixed on longitudinal and transverse axis lines where the white line of the right abdomen and the midpoint of the outer torso of mice were located. Dynamic ultrasound images were meticulously captured and archived for subsequent analysis. The thickness of the rectus abdominis muscle was determined through both longitudinal and transverse sonographic incisions.

### 2.12 Histopathology

The mice were euthanized by cervical dislocation. The right lung and diaphragm tissues were extracted, fixed in 4% paraformaldehyde, embedded in paraffin, and subjected to hematoxylin and eosin (H&E) staining for pathological examination. Following sectioning, the lung tissue and diaphragm were observed for any changes. Image‐Pro Plus 6.0 (Media Cybernetics, MD, USA) was employed to analyze the muscle fibers of the diaphragm and rectus abdominis, as well as the cross‐sectional area of the alveoli.

### 2.13 Enzyme‐Linked Immunosorbent Assay (ELISA)

ELISA was utilized to measure the expression of IL‐10, INF‐γ, and TNF‐α in BALF supernatant. Standard product dilution was performed with blank wells, standard product wells, and sample wells for measurement. Following the addition of samples and thorough mixing, the samples were incubated at 37°C for 60 min. Subsequently, the liquid was discarded, wells were rinsed 5 times, and then patted dry. Enzyme‐labeled reagent was added to all wells except the blank well, mixed well, and incubated at 37°C for 60 min. After washing the enzyme‐labeled plate, a color developer was added, and color development occurred at 37°C for 10 min. Termination fluid was added, and the absorbance (OD) of each well was measured at a wavelength of 450 nm after zeroing against the blank well. The standard curve's linear regression equation was determined based on the concentration of the standard product and its corresponding OD value. Subsequently, the sample's concentration was calculated using the regression equation based on the sample's OD value.

### 2.14 Immunofluorescence

Paraffin sections of the lung and bronchus were dewaxed with water. Antigen retrieval was performed using EDTA antigen retrieval buffer, cooled to room temperature, and washed with PBS on a decolorizing table for three sets of 5 min each. The sections were then dried slightly, BSA drops were added, and the samples were sealed at room temperature for 30 min. After discarding the sealing solution, the diluted primary antibody was added to a wet box at 4°C, incubated overnight, and rinsed with PBS three times for 5 min each. Following drying, a fluorescent secondary antibody was applied, incubated at room temperature for 1 h, and washed with PBS three times for 5 min each. The re‐stained nuclei were treated with DAPI at room temperature for 10 min and washed with PBS three times for 5 min each. Once the anti‐fluorescence quenching sealing tablets were applied, the localized expressions of MuRF‐1 (55456‐1‐AP, Proteintech, Wuhan, China) and IGF‐1 (28530‐1‐AP, Proteintech, Wuhan, China) proteins in the diaphragm and rectus abdominis were immediately observed under a fluorescence microscope.

### 2.15 Quantitative Real‐Time Polymerase Chain Reaction (PCR) Analysis

Total RNA was isolated from different diaphragmatic and rectus abdominalis samples by adding TRIzol (Invitrogen 15596018, California, USA). Single‐stranded cDNA was synthesized from 1 μg RNA using a reverse reaction kit (Invitrogen K1622, California, USA). Then use the SYBR green I Mater Mix kit (Invitrogen, Carlsbad, CA, USA), Real‐time quantitative reverse transcription polymerase chain reaction (qRT‐PCR) was performed on the Roche480II Real Time PCR System (Roche, Basel, Switzerland). Glyceraldehyde phosphate dehydrogenase (GAPDH) is used as an internal parameter for CasP3, Myod1 and Hspa5 to normalize comparisons. The sequence of primers used for PCR is shown in Table 1.

**Table 1:** Primers sequence for RT‐qPCR.

Gene	Forward primer	Reverse primer
*CasP3*	ATGGGAGCAAGTCAGTGGAC	CGTACCAGAGCGAGATGACA
*Myod1*	TCCGCTACATCGAAGGTCTG	CCGCTGTAATCCATCATGCC
*Hspa5*	CCTTGTGTTTGACCTGGGTG	CCATGACCCGCTGATCAAAG
*GAPDH*	AGGTCGGTGTGAACGGATTTG	TGTAGACCATGTAGTTGAGGTCA

### 2.16 Statistical Analysis

Data analysis was conducted the Statistical Package for the Social Sciences software, version 25.0 (SPSS Inc., Chicago, IL, USA). The staff who did not participate in group assignment was assigned to be responsible for statistics. Before statistical testing, the normality and homogeneity of variance of the data were assessed employing the Kolmogorov‐Smirnov test and Levene's test, respectively. Data conforming to a normal distribution were reported as the mean ± standard deviation (SD), whereas data exhibiting a skewed distribution were depicted as the median with interquartile range. Group comparisons were performed using one‐way analysis of variance (ANOVA), followed by post‐hoc analysis with the Bonferroni correction for multiple pairwise comparisons. In instances of heterogeneous variances, Dunnett's T3 test was implemented for group‐wise multiple comparisons. A threshold of *p* < 0.05 was set to denote statistical significance.

## 3 Results

### 3.1 All Four Stimulation Modalities Ameliorated Pulmonary and Diaphragm Function in COPD Mice

**Figure 3:**
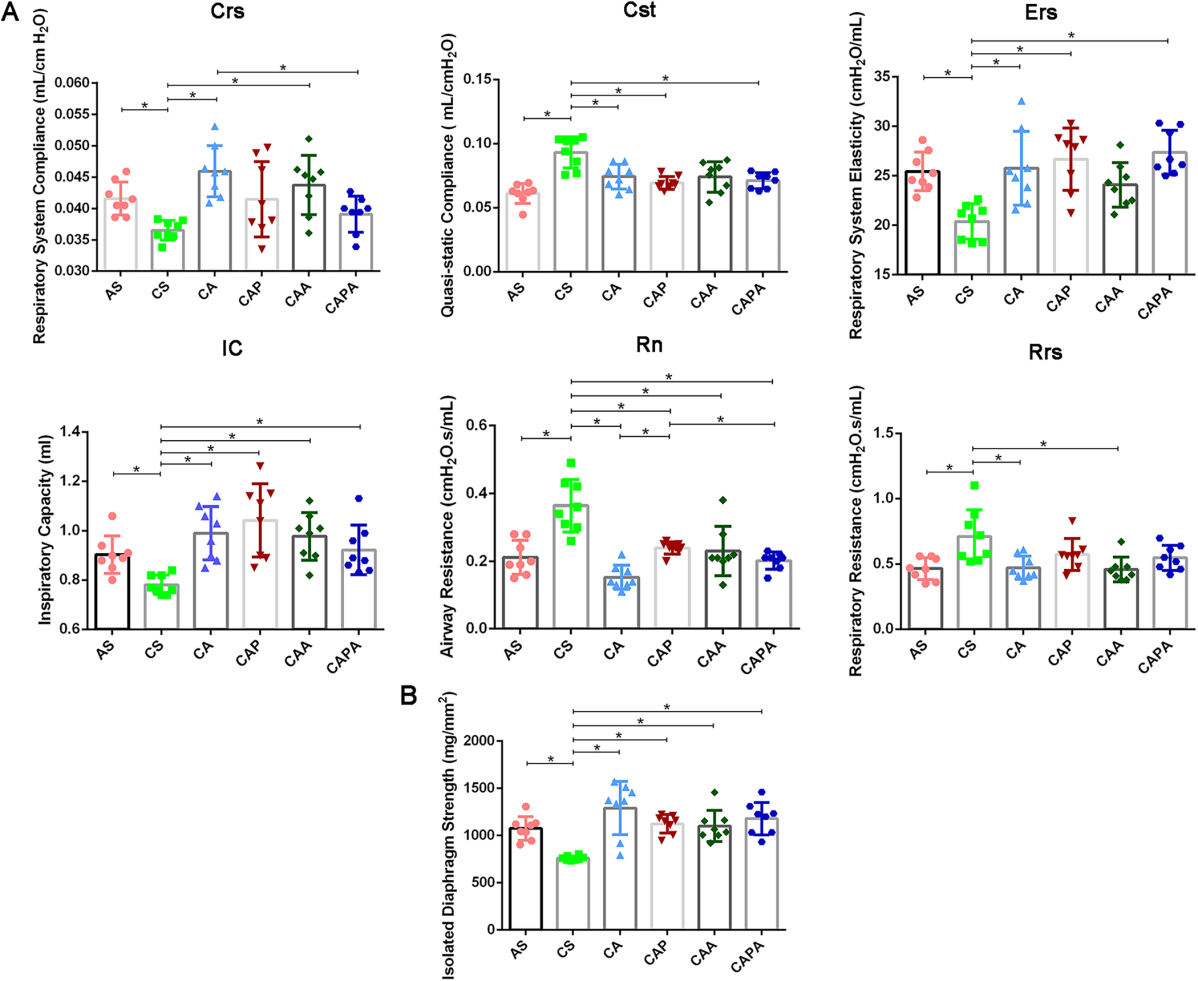
Interventions simulating the kinematic characteristics of Liuzijue Qigong improve pulmonary function and diaphragmatic contractility in COPD mice. Panels display the pulmonary function indicators (A) and diaphragmatic contractility (B) among different groups of mice. Data are expressed as mean ± standard deviation. AS, blank group; CA, aerobic exercise group; CAA, abdominal muscle stimulation group; CAP, pursed lip breathing group; CAPA, compound stimulation group; Crs, respiratory system compliance; CS, model group; Cst, quasi‐static compliance; Ers, respiratory system elasticity; IC, inspiratory capacity; Rn, airway resistance; Rrs, respiratory resistance.

The results of validation after replication of the COPD model were detailed in Figure S1. Exposure to cigarette smoke in conjunction with intra‐airway LPS induced a pronounced deterioration in respiratory function in mice, characterized by elevated Cst, Rn, and Rrs values, alongside diminished Crs, Ers, and IC (*p* < 0.05) (Figure 3). Postintervention, low‐intensity aerobic exercise and the integration of abdominal muscle stimulation significantly augmented Crs and markedly reduced Rrs (*p* < 0.05). Notably, the static compliance (Cst) of CA, CAP and CAPA decreased, while the elasticity (Ers) of lung tissue was enhanced (*p* < 0.05). Each of the Liuzijue kinematic characteristic‐based stimulations significantly elevated IC and reduced Rn (*p* < 0.05). Of particular note, the CA and CAPA interventions demonstrated superior efficacy in attenuating main airway resistance (Rn) compared to the CAP intervention (*p* < 0.05).

The 40‐day modeling process was observed to compromise the contractility of diaphragmatic muscle strips following in vitro electrical stimulation (*p* < 0.05). Postintervention, all four exercise modalities exerted positive effects on enhancing the in vitro contractile capacity of the diaphragmatic muscle strips (*p* < 0.05). No significant differences were detected among the various exercise modalities in their ability to improve diaphragmatic muscle function.

### 3.2 Pursed‐Lip Stimulation Had Limited Positive Effects on Rectus Abdominis Compared to the Other Modalities of Stimulation

**Figure 4:**
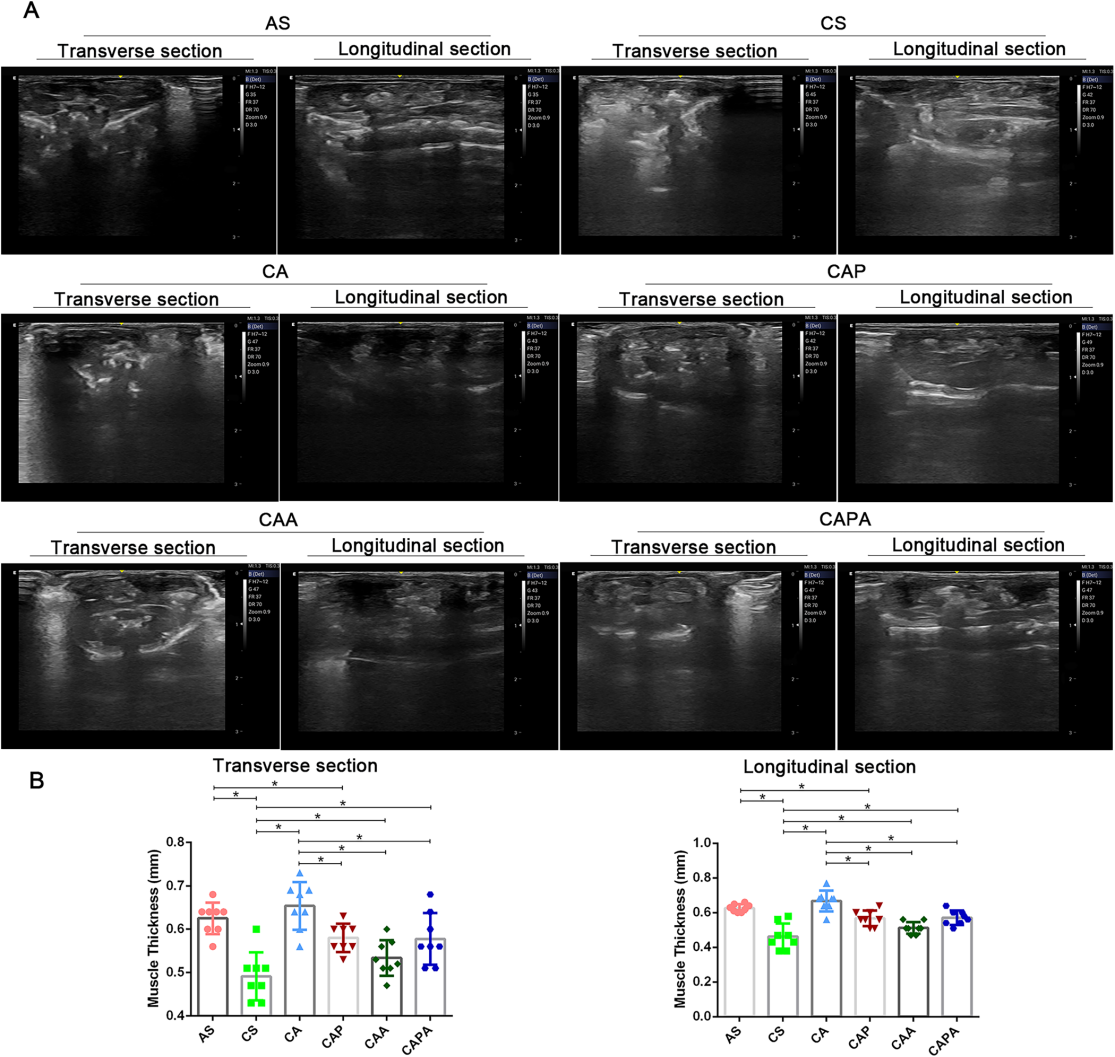
The impact of interventions emulating the kinematic features of Liuzijue on the thickness of the rectus abdominis muscle in COPD mice. Panel (A) displays representative ultrasonic images of the rectus abdominis muscle in mice across different experimental groups; Panel (B) illustrates the comparative analysis of the muscle thickness measured by ultrasound. Data are expressed as mean ± standard deviation. AS, blank group; CS, model group; CA, aerobic exercise group; CAP, pursed lip breathing group; CAA, abdominal muscle stimulation group; CAPA, compound stimulation group.

Ultrasound imaging assessments of the rectus abdominis muscle, in both transverse and longitudinal sections, demonstrated that all types of stimulation, with the exception of pursed‐lip breathing, positively influenced muscle thickness (Figure 4). Throughout the 40‐day modeling period, a significant reduction in the thickness of the rectus abdominis muscle was observed in both transverse and longitudinal sections (*p* < 0.05). Postintervention, mice in the other three groups receiving various exercise interventions, excluding the CAP group, displayed a significant increase in abdominal muscle thickness (*p* < 0.05). Notably, low‐intensity aerobic exercise led to a more pronounced enhancement in abdominal muscle thickness compared to other combined stimulation interventions (*p* < 0.05). The impact of combining pursed‐lip respiratory stimulation with aerobic exercise on abdominal muscle thickness did not yield conclusive results.

### 3.3 Low‐Intensity Aerobic Exercise and Abdominal Stimulation Improved the Respiratory Muscle Structure of COPD Mice

Mice subjected to ambient air conditions displayed distinct pulmonary tissue architecture, with uniform alveolar spacing and preserved alveolar dimensions. In contrast, exposure to cigarette smoke and LPS resulted in notable histopathological alterations, including damaged alveolar septa, enlargement of some alveoli, and a disrupted hierarchical structure of the terminal respiratory tract. Additionally, an influx of inflammatory cells and thickening of the lung interstitial tissue were observed in localized regions. Interestingly, the exercise interventions, incorporating diverse kinematic patterns inspired by Liuzijue, exerted limited effects on the alveolar structure of the mice. The alveolar structures in all exercise groups displayed structural characteristics similar to those of mice exposed to cigarette smoke. Moreover, a comparative analysis of the alveolar cross‐sectional area within randomly selected fields indicated that the majority of exercise interventions had minimal influence on the phenomena of alveolar fusion and dilation, although mice in CAA had smaller alveolar cross‐sectional area compared to those in the CS with larger alveolar area.

**Figure 5:**
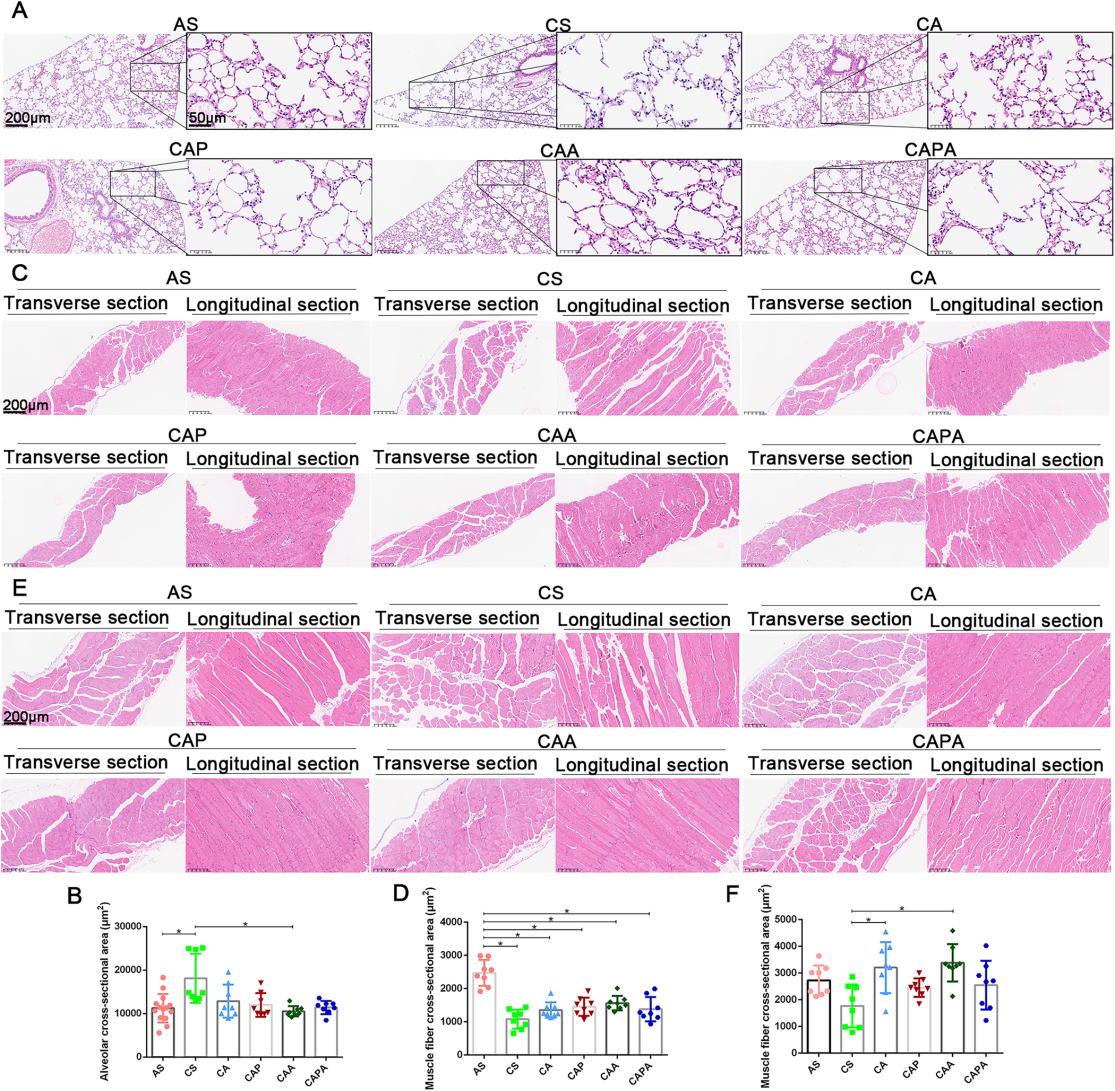
Morphological changes in respiratory muscles of COPD mice induced by interventions simulating the kinematic characteristics of Liuzijue. Panel (A) presents the structure of lung tissue from different groups of mice, with the scale bar set at 200 μm for the lower magnification and 50 μm for the higher magnification views; Panel (B) compares the cross‐sectional areas of alveoli among the various groups. Panel (C) shows H&E staining of diaphragmatic tissue with a scale bar of 200 μm; Panel (D) illustrates the comparison of cross‐sectional areas of diaphragmatic muscle fibers. Panel (E) depicts H&E staining of the rectus abdominis muscle with a scale bar of 200 μm; Panel (F) presents the comparison of cross‐sectional areas of muscle fibers in the rectus abdominis. Data are expressed as mean ± standard deviation. AS, blank group; CS, model group; CA, aerobic exercise group; CAP, pursed lip breathing group; CAA, abdominal muscle stimulation group; CAPA, compound stimulation group.

The synergistic exposure to cigarette smoke and LPS was observed to induce deleterious effects on the respiratory muscle architecture in mice, notably impacting key muscles such as the diaphragm and rectus abdominis. Histological examination of tissue sections demonstrated a diminished density of muscle fibers in both transverse and longitudinal orientations, suggestive of respiratory muscle atrophy subsequent to the modeling process. This observation was corroborated by quantitative assessments, which disclosed a statistically significant reduction in the cross‐sectional area of diaphragmatic muscle fibers in cigarette smoke‐exposed mice (CS) as compared to their healthy counterparts (*p* < 0.05). Intriguingly, the implementation of exercise intervention post‐modeling exerted salutary effects on the atrophic muscular structures in the mice. This was evidenced by a more condensed arrangement of muscle fibers within the visual field. Specifically, the beneficial impact was pronounced following low‐intensity aerobic exercise coupled with abdominal stimulation. The comparative analyses of the cross‐sectional area of abdominal muscle fibers in CA and CAA groups compared to CS substantiated these positive outcomes (*p* < 0.05) (Figure 5).

### 3.4 All Simulated Exercise Modalities Inhibited the Airway Inflammation

**Figure 6:**
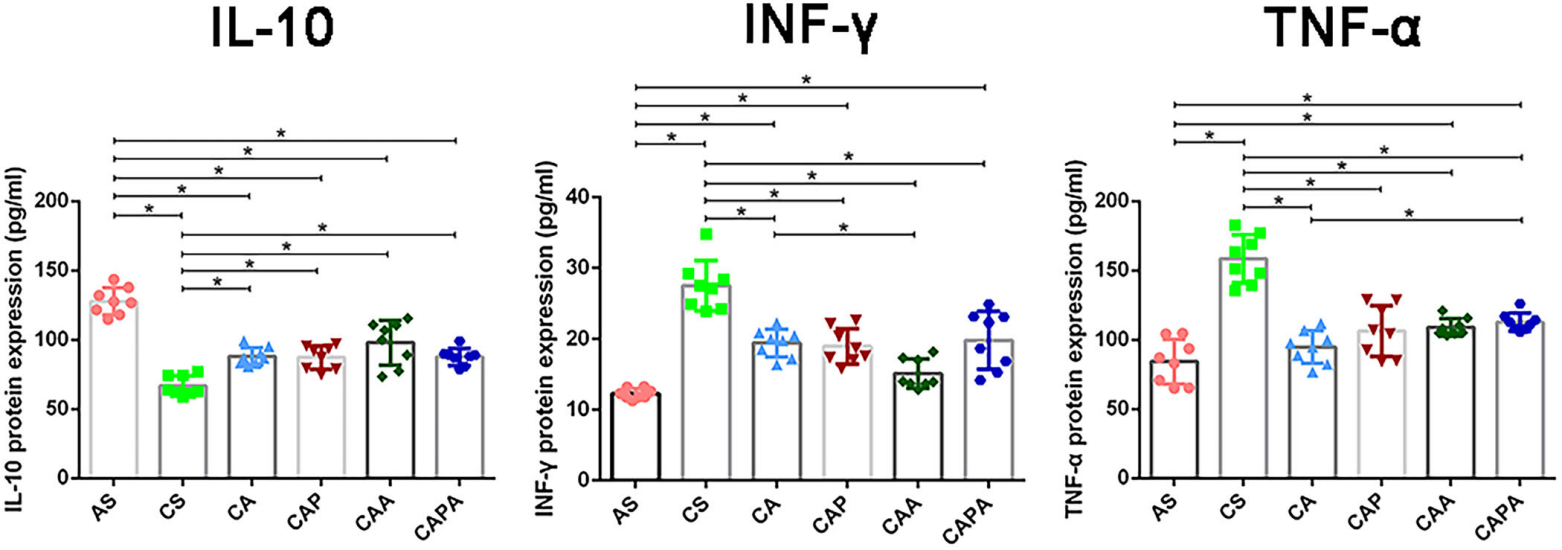
Inhibition of airway inflammation in COPD mice by interventions simulating the kinematic characteristics of Liuzijue. Data are expressed as mean ± standard deviation. AS, blank group; CA, aerobic exercise group; CAA, abdominal muscle stimulation group; CAP, pursed lip breathing group; CAPA, compound stimulation group; CS, model group; IL, interleukin; INF, interferon; TNF, tumor necrosis factor.

The 40‐day modeling process resulted in a pronounced inflammation in the airways of mice, evidenced by heightened expression of pro‐inflammatory cytokines INF‐γ and TNF‐α. Interestingly, this inflammatory milieu was accompanied by a decreased level of IL‐10, a cytokine with established anti‐inflammatory properties. Notably, pro‐inflammatory cytokines remained significantly downregulated after a 9‐week period of exercise cessation, and the anti‐inflammatory properties of the four interventions also have been shown (Figure 6). Although all exercise groups increased IL‐10 expression and the complexity of exercise regimens varied among groups, there was no significant difference in enhancing anti‐inflammatory cytokines (IL‐10) between the CA, CAP, CAA, and CAPA groups. On the contrary, when comparing the CA group with the CAP and CAPA groups, there was a significant difference in the expression of INF‐γ and TNF‐α (*p* < 0.05), indicating that the addition of stimuli may have varying effects on the expression of different inflammatory cytokines.

### 3.5 All Simulated Exercise Modalities Regulated the Balance Between Muscle Synthesis and Degradation

**Figure 7:**
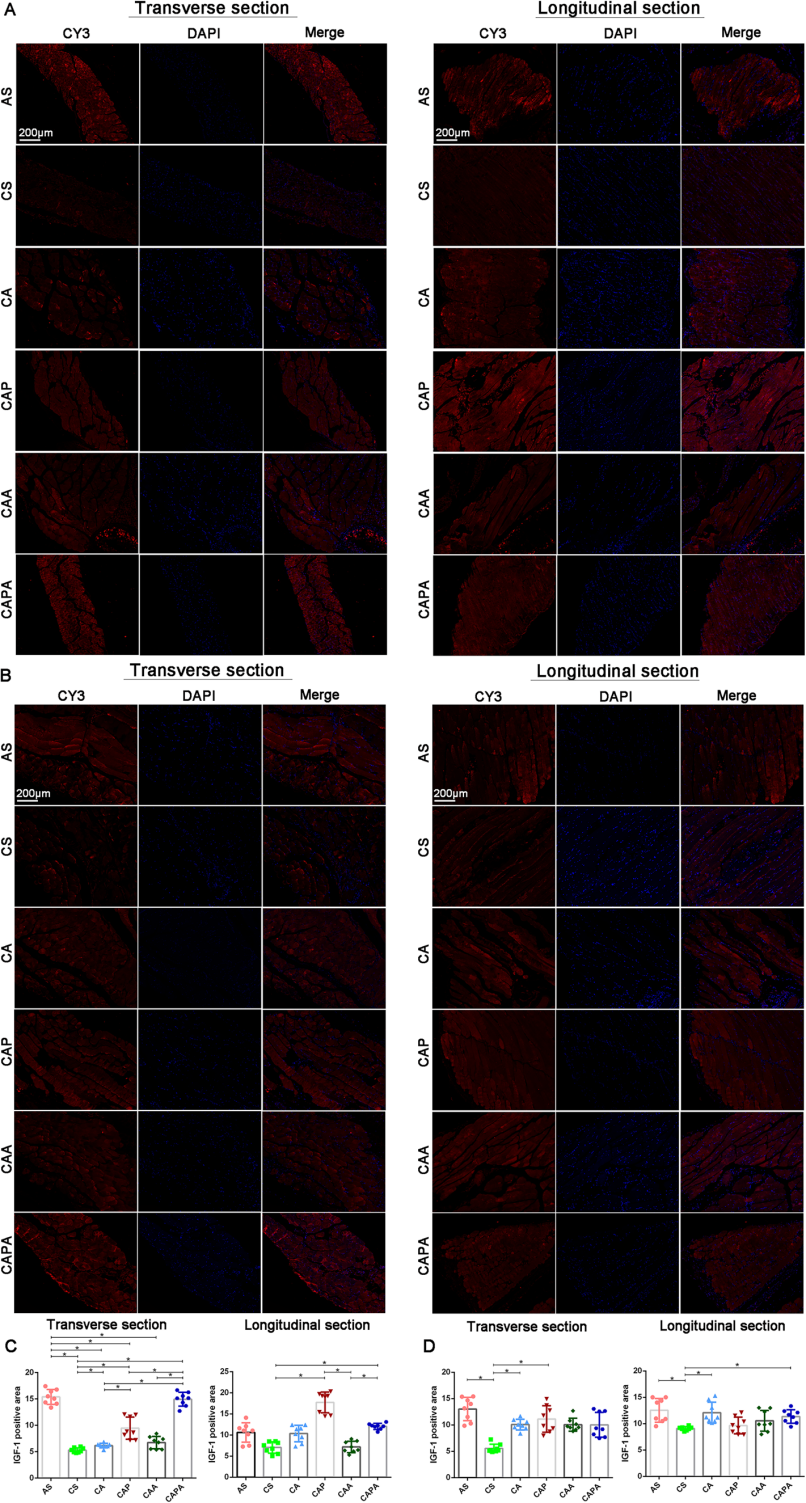
The promotional effects of interventions derived from the kinematic characteristics of Liuzijue on IGF‐1 expression in respiratory muscles of COPD mice. Panel (A) shows the expression of IGF‐1 in the diaphragm across different groups; Panel (B) presents the expression of IGF‐1 in the rectus abdominis muscle. Panels (C) and (D) depict comparative results of IGF‐1 expression in the diaphragm and rectus abdominis, respectively. Data are expressed as mean ± standard deviation. Bar = 200 μm. AS, blank group; CS, model group; CA, aerobic exercise group; CAP, pursed lip breathing group; CAA, abdominal muscle stimulation group; CAPA, compound stimulation group.

**Figure 8:**
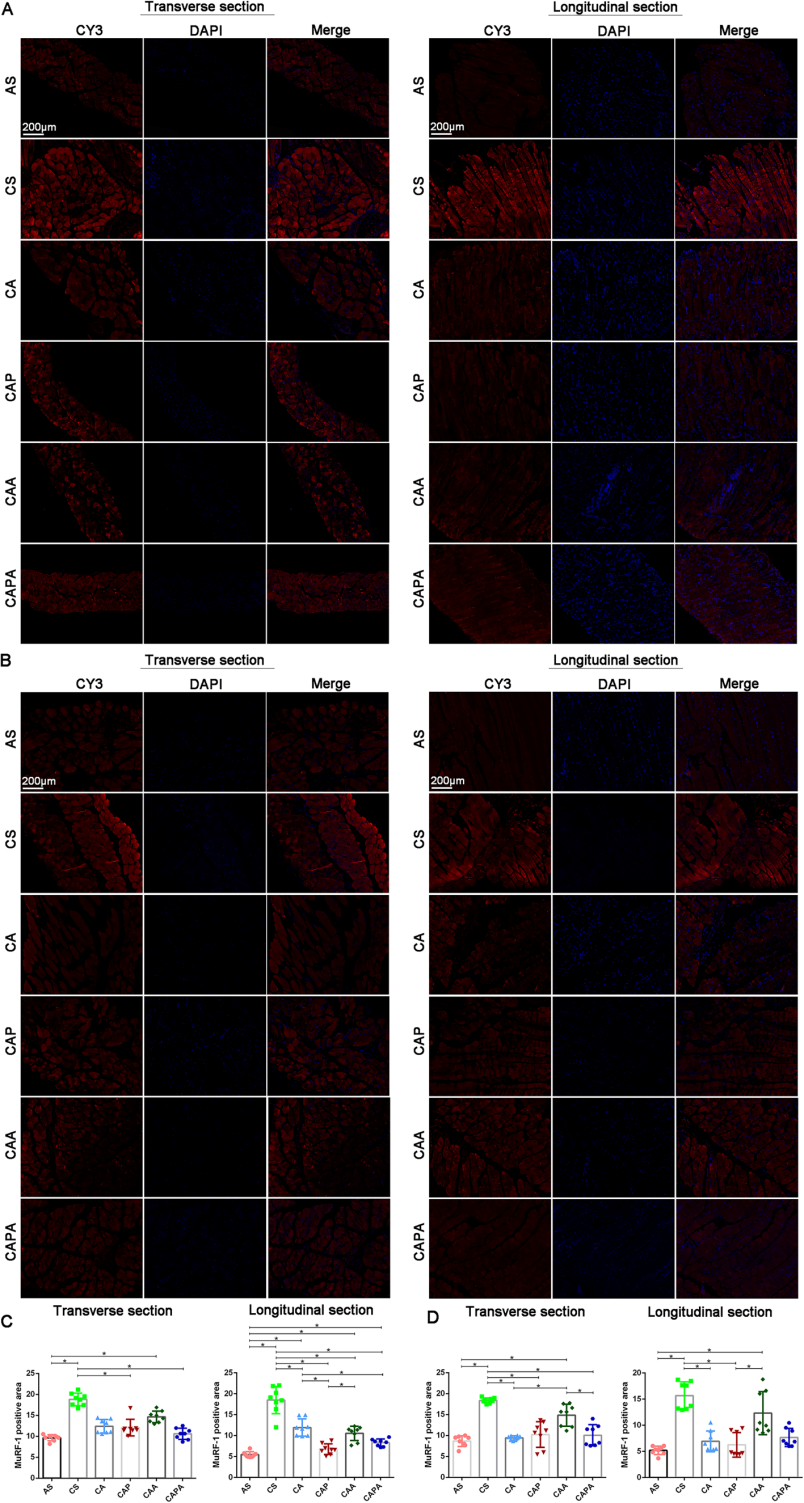
The impact of interventions based on the kinematic characteristics of Liuzijue on MuRF‐1 expression in respiratory muscles of COPD mice. Panel (A) illustrates the MuRF‐1 expression in the diaphragm among various groups; Panel (B) shows the MuRF‐1 expression in the rectus abdominis muscle. Comparative analysis of MuRF‐1 expression in the diaphragm is presented in Panel (C), and in the rectus abdominis in Panel (D). Data are expressed as mean ± standard deviation. Bar = 200 μm. AS, blank group; CS, model group; CA, aerobic exercise group; CAP, pursed lip breathing group; CAA, abdominal muscle stimulation group; CAPA, compound stimulation group.

Immunofluorescence assessments revealed that four types of stimulation, inspired by the kinematics of Liuzijue, exerted positive effects on the homeostatic balance of muscle protein synthesis and degradation in both the diaphragm and rectus abdominis of the mice (Figures 7 and 8). Specifically, IGF‐1 expression levels in the diaphragm and rectus abdominis of mice exposed to smoke were significantly diminished relative to healthy controls (*p* < 0.05). On the contrary, the expression of IGF‐1 in the diaphragm and rectus abdominis of mice increased significantly after intervention. Post‐intervention, however, a marked increase in IGF‐1 expression was observed in these muscle‐trained groups. Moreover, the diaphragmatic tissue of mice that underwent the lip‐pursed breathing interventions, CAP and CAPA, demonstrated a significantly greater enhancement in IGF‐1 expression compared to other intervention methods (*p* < 0.05). Conversely, MuRF‐1 expression levels in the diaphragm and rectus abdominis of CS mice were significantly elevated above those of other groups, with a notable decreasing trend following the interventions. It is noteworthy that MuRF‐1 expression in the diaphragm of mice post lip‐pursed breathing intervention (CAP and CAPA) was significantly reduced compared to the COPD mice (*p* < 0.05). The abdominal muscle stimulation did not significantly influence the regulatory dynamics of IGF‐1 and MuRF‐1 in the respiratory muscles.

**Figure 9:**
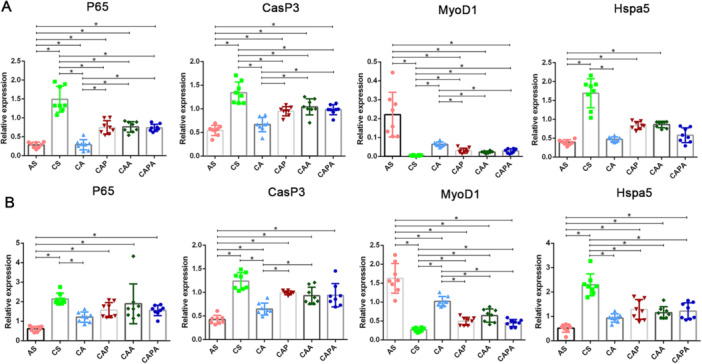
Effects of interventions simulating the kinematic characteristics of Liuzijue on the expression of inflammatory, autophagic, growth and stress factors in respiratory muscles of COPD mice. Panel (A) presents the comparative transcription levels of P65, CasP3, MyoD1 and Hspa5 in the diaphragm of different groups of mice; Panel (B) presents the comparative transcription levels of P65, CasP3, MyoD1 and Hspa5 in the rectus abdominis of different groups of mice. Data are expressed as mean ± standard deviation. AS, blank group; CS, model group; CA, aerobic exercise group; CAP, pursed lip breathing group; CAA, abdominal muscle stimulation group; CAPA, compound stimulation group.

The results of RT‐PCR findings substantiated the regulatory influences of the four intervention strategies on the equilibrium between muscle growth and degradation in our study (Figure 9). The mRNA expression patterns of P65 and CasP3 in the diaphragm and rectus abdominis of cigarette smoke‐exposed (CS) mice were congruent, exhibiting significantly higher levels compared to healthy mice (*p* < 0.05). This elevation indicates a robust activation of inflammatory and autophagic pathways in the respiratory muscles of COPD mice following exposure to cigarette smoke and LPS. In tandem with the degree of airway inflammation, all interventions significantly suppressed P65 expression in the diaphragm (*p* < 0.05), which corresponded to a reduction in autophagy levels. The majority of intervention methods effectively restored the diminished MyoD1 expression levels in the diaphragm and rectus abdominis of COPD mice (*p* < 0.05). Notably, low‐intensity aerobic exercise, when implemented in isolation, showed a distinct ability to augment MyoD1 expression within the aforementioned muscular tissues.

Furthermore, the study disclosed that the expression levels of Hspa5 in the diaphragm and rectus abdominis of mice exposed to cigarette smoke were markedly elevated in comparison to other study groups, highlighting the model's provocative effect on endoplasmic reticulum (ER) stress within murine muscle cells and affirming the effectiveness of the four exercise interventions in mitigating ER stress levels. It is of particular interest that the CA group, which received simpler stimulation, displayed a significantly more pronounced inhibitory effect on Hspa5 expression in the diaphragm and on P65 and CasP3 in the rectus abdominis, relative to other intervention groups. This finding suggests that complex stimulation paradigms may not inherently offer superior benefits in alleviating ER stress within the respiratory musculature of mice.

## 4 Discussion

### 4.1 Kinematic Simulation Approach for Liuzijue

Liuzijue, a traditional Chinese Qigong practice, is recognized as a significant nonpharmacological intervention for airway management in COPD patients during remission phases. Its positive impact on respiratory function, exercise capacity, and the overall quality of life in COPD patients has been well‐documented. However, research on the therapeutic mechanism of traditional Chinese medicine, specifically Liuzijue, remains in the nascent stages of research. Despite its simplified movements relative to other forms such as Taichi and Baduanjin, replicating the kinematic patterns of Liuzijue in animal models presents unique challenges. Current research leverages theoretical analysis to dissect the kinematic characteristics of Liuzijue [11]. Initially, it is noted that Liuzijue resembles a form of low‐intensity aerobic exercise. To date, there has been a paucity of direct research on heart rate variability during the practice of Liuzijue. In the absence of direct evidence to confirm the exercise intensity during its practice, the study draws parallels with Taichi, a practice sharing similar attributes with Liuzijue. Taichi practice is reported to involve heart rate variability at 58% of heart rate reserve, with oxygen uptake (VO_2_) at 55% of peak oxygen uptake (VO_2_ peak) [20]. Given that the movement amplitude of movement in Liuzijue is less pronounced than that of Taichi, it is hypothesized that the exercise intensity of Liuzijue is more aligned with low‐intensity aerobic activity. Secondly, each of the six foundational movements in Liuzijue culminates in pursed‐lip exhalation accompanied by vocalization. This process serves an exercise function for the respiratory muscles, similar to that of pursed‐lip breathing [21]. Electrical impedance tomography has shown that pursed‐lip breathing increases the depth of inhalation and the ratio of exhalation time, enhancing exhalation flow uniformity and respiratory stability beyond that of normal breathing conditions [22]. In our experiment, kinesio tape was applied around the mice's lips to restrict nasal and oral airflow during respiration; however, the specific gas flow parameters could not be assessed under the current experimental setup. Thirdly, Liuzijue emphasizes abdominal breathing, increasing respiratory depth, which embodies the traditional Chinese medical concept of “Qi sinking to the Dantian.” Abdominal breathing is extensively utilized in the pulmonary rehabilitation of COPD patients and has demonstrated positive effects even during acute episodes [23]. We induced abdominal breathing in mice by applying kinesiology tape with varying tensions on the chest and abdomen, thereby enhancing the respiratory function of the model animals. Therefore, grounded in the theoretical analysis of Liuzijue's kinematic traits, integrates the practice's principal features ‐ namely, augmenting the respiratory load in the distal airways, effectively activating abdominal muscles, and incorporating low‐intensity aerobic exercise ‐ into a COPD mouse model. This study aims to develop an exercise model intervention reflective of Liuzijue's kinematic characteristics, offering insights and a reference framework for investigating its therapeutic mechanisms.

### 4.2 Pulmonary Function Improvements

In the present study, a COPD animal model was established using a combination of whole‐body cigarette smoke exposure and intra‐airway instillation of LPS. Consistent with the majority of studies reported in the literature [24, 25, 26], the decline in respiratory function indicators in cigarette smoke exposed mice after a 40‐day modeling process provided evidence for the successful replication of the COPD animal model. The abnormalities in Crs, Cst, and Ers in CS mice may be associated with hyperinflation within the thoracic cavity and modifications in collagen and elastin fibers within the extracellular matrix of lung tissue in the COPD model mice [27]. Additionally, CS mice demonstrated elevated airway resistance, including Rn and Rrs. Furthermore, in this study, the ameliorative effects of various forms of exercise training on the respiratory function of COPD mice were similar to previous research reports [28, 29]. The lung function improvement in the CA group was the most pronounced among all groups, demonstrating that low‐intensity aerobic exercise possesses a high degree of maturity in animal experimental interventions. The marked enhancement of Cst and Ers in the CAP and CAPA groups implies that increasing respiratory resistance around the lips may positively influence lung tissue elasticity. Although existing evidence confirms the role of pursed‐lip breathing in improving lung function in COPD patients [30, 31], the direct impact of pursed‐lip breathing on lung tissue compliance or elasticity warrants further investigation, given the inconsistencies between clinical studies and animal model data. Furthermore, this study confirmed the positive influence of abdominal breathing on the respiratory function of COPD mice, particularly regarding airway resistance. This finding is significant despite the inability to replicate an autonomous mouse abdominal breathing pattern. The beneficial effects of abdominal breathing on the ventilation status of COPD patients have long been acknowledged [32]. It has been found that most COPD patients have reduced mobility of the chest and abdominal walls, which is correlated with forced vital capacity (FVC); fortunately, compared with the mobility of the chest wall, the mobility of the abdominal wall is relatively preserved [33], providing a basis for the greater recruitment of the diaphragm and abdominal muscles during respiratory movements. Lastly, all intervention methods increased IC of the mice, indicating the positive impact of exercise training on the lung volume indicators of COPD mice, which is consistent with results obtained in our previous studies [19].

In the analysis of lung tissue structure the cohorts of mice, our pathological examination confirmed that a 40‐day regimen of cigarette smoke exposure integrated with LPS instillation effectively replicated a COPD mouse model. This affirmation was based on the observable alveolar enlargement and interstitial proliferation within certain visual fields, which are considered direct contributors to the diminished respiratory function characteristic of COPD mice. However, following a 9‐week intervention with diverse exercise modalities, the structural changes in the lung tissue of the majority of mice were not markedly pronounced, with the destruction and fusion of alveolar structures still discernible in some areas. An ensuing comparison of the alveolar cross‐sectional area further corroborated these findings, aligning with the outcomes of our prior research [19]. Nevertheless, the existing literature has indicated that exercise possesses the capacity to mitigate cigarette‐induced emphysematous lung damage, as evidenced by a reduction in enlarged alveoli, collagen fiber deposition [34], and an increased in alveolar linear intercept [28]. The divergence in our results may be imputed to the variability in exercise prescription parameters, given that the duration of exercise in the referenced studies typically surpassed the 9‐week threshold employed in this investigation. Similarly, the differences in exercise stimulation parameters in this study also led to distinct impacts on the lung tissue of the model mice: the alveolar cross‐sectional area in the CAA group was notably reduced in comparison to the CS group, suggesting that the amalgamation of aerobic exercise and abdominal breathing stimulation exerts a beneficial influence on the emphysematous damage in COPD mice. Upon amalgamating the findings of this study, it is postulated that the salutary effects of Liuzijue on the structural enhancement of COPD‐affected lung tissue may be contingent upon the simulation of the exercise's kinematic characteristics through specific stimulation forms and parameters. Consequently, further exploration into the parameters that emulate the kinematic attributes of Liuzijue should remain a focal point of interest for future research.

### 4.3 Respiratory Muscle Adaptation

The core kinematic features of Liuzijue are hypothesized to ameliorate the respiratory status of COPD mice by enhancing the functionality and structure of respiratory muscles. Considering the integrated assessment of respiratory mechanics indices and airway structure, the intervention mimicking the kinematic characteristics of Liuzijue may not exert a direct effect on the airways themselves in enhancing the respiratory function of mice induced by cigarette smoke. A comparison of multiple respiratory mechanics indices across the four intervention groups revealed no significant differences among them. This could be attributed to the fact that the exercise modes of CAA, CAP, and CAPA are all based on swimming, with additional factors having a less pronounced impact on respiratory mechanics than swimming itself [35]. Specifically, after intervention, the IC of CS mice showed significant improvement, suggesting a notable enhancement in tidal volume or inspiratory reserve volume [36]. Given the nonsignificant differences in the terminal airway structures of the mice, particularly in alveolar, the increase in inspiratory capacity in the model mice after intervention primarily relied on the force generated by the active contraction of the inspiratory muscles [37], a viewpoint supported by the results of diaphragmatic contractility and rectus abdominis thickness. The improvement of IC may be related to an increase in tidal volume or inspiratory reserve volume; however, as these indices were not collected in the present study, further research is needed to confirm this. Airway resistance is the frictional force that must be overcome during respiration, resulting from the anatomical structure of the airways and the tissue viscous resistance provided by the lungs and adjacent tissues and organs [38]. As previously mentioned, the increase in the Rn value in CS mice is mainly related to the narrowing of the airways in CS mice. Reports have shown that both airway smooth muscle hyperplasia and thickening of the extracellular matrix affect the luminal diameter of the airways and directly increase airway resistance [39, 40]. Exercise training has a significant inhibitory effect on airway smooth muscle hyperplasia [41], which may be one of the pathways by which the exercise groups reduce Rn values. However, our study did not find representative sections in airway structure that indicate the actual impact of the four intervention methods on airway lumen. Furthermore, Rrs is associated with the frictional resistance of airway and chest wall movements, particularly with inflammatory responses within the airways, which can elevate Rrs [40]. Lastly, the negative correlation between Crs and Rn, consistent with most results, leads us to speculate, in conjunction with the results of the Ers in this study, that the model replication process may cause changes in collagen and elastic fibers in the extracellular matrix components of lung tissue. However, as this study did not perform special staining for fibrous tissues, this viewpoint requires further validation. Additionally, whether the difference in the expression trends of Cst and Crs in CS mice is related to the intervention of airway resistance needs to be explored in subsequent studies.

Alternatively, corroborated by the findings on diaphragmatic strength and abdominal muscle thickness, we surmise that the beneficial impact of exercise training on the respiratory function of COPD mice is likely associated with the augmented function of respiratory muscles, notwithstanding the diversity in exercise stimulation modalities applied. Our study's results indicated that all stimulation methods effectively enhanced diaphragmatic function in COPD mice, with abdominal stimulation positively influencing the thickness of the rectus abdominis, as evidenced by ultrasound imaging. Additionally, H&E staining of the diaphragm and rectus abdominis revealed that the interventions employed in this study impart varying degrees of beneficial effects on the muscular structures in the thoracic and abdominal regions. Exercise training, a cardinal component of pulmonary rehabilitation, has been validated by extensive research to enhance the primary respiratory muscles in COPD patients [42, 43]. Nonetheless, discrepancies exist in the ameliorative effects of different exercise stimulation forms on respiratory muscle function in COPD [44]. This study aligns with prior research affirming that aerobic exercise significantly improves lung function indicators that reflect respiratory muscle capacity and clinical presentations such as dyspnea [45, 46], with affirmative impacts observed even in individuals with advanced stages of the disease [47]. Moreover, the simulation of pursed‐lip breathing in this study produced outcomes akin to those in clinical studies [48], demonstrating a significant impact of acute pursed‐lip breathing on respiratory rate and minute ventilation in COPD patients [49]. Pursed‐lip breathing fosters a slower, more profound breathing pattern during both rest and exercise, and the improvement in respiratory status post intervention is intricately linked to the recruitment status of respiratory muscles during the training, underscoring that the extent of respiratory muscle engagement is a pivotal determinant of pursed‐lip breathing's effectiveness [50]. Moreover, the analysis of the cross‐sectional area of the rectus abdominis muscle fibers indicates that the simulated abdominal breathing stimulation has a more pronounced impact on the structure of the rectus abdominis in COPD mice, given the higher degree of abdominal activity in this breathing pattern [51]. However, compared to the positive effects achieved with combined pursed‐lip breathing and abdominal breathing interventions in COPD patients [30], the composite intervention in this study did not yield the expected outcomes, suggesting that the application of composite intervention stimuli in experimental animals may not result in a simple additive effect.

### 4.4 Anti‐Inflammatory and Molecular Pathways

Our findings suggest that the primary kinematic effects of the Liuzijue may ameliorate the respiratory state in COPD by influencing factors such as inflammation levels, muscle autophagy, proteolysis, and muscle growth. All forms of stimulation exerted an inhibitory effect on the inflammatory levels within the airways of COPD mice. It has been demonstrated that a single bout of incremental exercise can induce an anti‐inflammatory effect on the airways of COPD patients [52]. Regular, moderate‐intensity exercise training has also shown a positive effect in reducing serum levels of C‐reactive protein and IL‐8 in the pulmonary rehabilitation management process of COPD patients [53]. Existing evidence has established that pulmonary inflammation in COPD patients is a key factor in inducing extrapulmonary damage, including respiratory muscle dysfunction [54]. Smoking induces recurrent inflammation, followed by the chronic and progressive activation of the immune system. In the pulmonary system of smokers, oxidative stress, cellular damage, and chronic activation of pattern recognition receptors have been described, leading to the translocation of NF‐κB, the release of pro‐inflammatory cytokines, chemokines, matrix metalloproteinases, and damage‐associated molecular patterns. Concurrently, smoke pollutants directly traverse the alveolar‐capillary interface, diffusing through the systemic bloodstream to target different organs and cause damage. In muscle tissue, the inflammatory process activates catabolic signaling pathways, leading to muscle atrophy and sarcopenia. Long‐term exposure to cigarette smoke results in a reduction of type I fiber percentage, a decrease in muscle fiber cross‐sectional area, increased activity of glycolytic enzymes, and reduced muscle oxidative activity [55]. Furthermore, the activation of inflammatory signals also induces signs of apoptosis and autophagy, as well as an imbalance between protein synthesis and degradation [56]. This includes an increase in the ubiquitination of tissue proteins, indicated by increased activity of the E3 ubiquitin ligases atrogin‐1 and MuRF1, accompanied by a reduction in the synthesis of key factors for protein synthesis such as IGF‐1 and MyoD1 [57]. Our study also shows similar results. Moreover, the inhibitory effect of appropriate exercise on the airway inflammation levels in COPD may be the primary pathway of the pulmonary rehabilitation effect of exercise. In this study, the four intervention forms established based on the main kinematic characteristics of Liuzijue demonstrated an inhibitory effect on the transcriptional levels of key factors of inflammation and apoptosis in the diaphragm of mice exposed to smoke, and significantly enhanced the protein synthesis level in the diaphragm muscle. Meanwhile, there were still differences between the three groups of composite stimulated mice (CAP, CAA, and CAPA) compared with simple aerobic exercise stimulation, although no significant differences were observed among the three groups. We believe that these four forms of stimulation based on non‐weight‐bearing swimming effectively stimulate the diaphragm and regulate related factors; this is consistent with previous research results as an intervention method [58]. However, except for CA, the inhibitory effects of the other three groups on the levels of inflammation and apoptosis in the rectus abdominis of mice were not obvious, which may suggest that the other three stimulation methods improve the structure and function of the rectus abdominis through other pathways, such as by enhancing the expression level of MyoD1. This hypothesis still needs to be supported by subsequent research.

It is noteworthy that our study identified four stimulation methods based on the kinematic characteristics of the Liuzijue Qigong. Regardless of the parameter assessed‐pulmonary function, airway inflammation, or the expression of key factors in inflammation and apoptosis within respiratory muscles‐the simple aerobic exercise stimulation demonstrated superiority. Considering the expression patterns of Hspa5 in the diaphragm and rectus abdominis of mice, we posit that the intervention effects in the animal model do not necessarily improve with the complexity of the intervention stimuli. Hspa5 is involved in the majority of the body's stress responses [59]. Although we endeavored to minimize the negative impact of the intervention on the mice during the process, it must be acknowledged that compared to swimming, the other three stimulation methods may elicit a more intense stress response in the mice. This could be a potential reason why the low‐intensity aerobic exercise intervention method achieved a relatively prominent effect in our study. The expression of Hspa5 is linearly related to the timing and extent of endoplasmic reticulum stress [60], and its expression trend among the groups may also be associated with the exercise's ability to suppress Hspa5 expression, reduce inflammatory responses, and achieve rehabilitative effects. However, synthesizing all the results from this study, we believe that the exercise stimulation methods simulated based on the kinematic characteristics of Liuzijue still possess the fundamental attributes of Liuzijue. This is evidenced by the impact of these stimulation methods on various indicators in COPD mice, including respiratory function, respiratory muscle strength, respiratory muscle structure, and levels of inflammation.

### 4.5 Limitations and Future Directions

This study provides the first methodological framework for simulating Liuzijue's kinematic characteristics in an animal model, addressing a critical gap in traditional nonpharmacological intervention research. By innovatively combining kinesio‐taping techniques with aerobic exercise paradigms, we established a translatable approach to investigate respiratory biomechanics in COPD pathophysiology. The integrated assessment of pulmonary function, respiratory muscle dynamics, and molecular pathways comprehensively validates the model's physiological relevance. However, our study has several limitations. Firstly, as a murine model, our findings cannot be directly extrapolated to humans. Caution is warranted when interpreting the results due to the distinct differences in breathing patterns and species‐specific physiological traits between humans and mice. Secondly, kinesio‐taping only partially mimics pursed‐lip/abdominal breathing and cannot replicate the conscious breath control central to Liuzijue's mind‐body integration. Neuromuscular coordination during traditional practice remains unmodelled. Thirdly, the limb movements in Liuzijue differ from the swimming motion of mice, leading to differences in target muscle stimulation. Moreover, the experimental conditions at present do not allow for precise control of various parameters in the model establishment process, such as the respiratory airflow through the nasal and oral cavities of mice after simulating pursed‐lip breathing. Future research will explore the impact of interventions mimicking Liuzijue's kinematic characteristics on ventilatory mechanics. Lastly, the criteria for gauging the success of the model still require support from a richer array of detection methods, which need to be addressed in subsequent studies.

Looking forward, this study paves the way for three key advancements. First, it provides a foundation for refining stimulation parameters (e.g., duration‐intensity relationships) to enhance human‐to‐animal translational fidelity. Second, it suggests exploring systemic immunomodulatory effects beyond the respiratory system. Third, it highlights the potential for clinical translation through biomarker‐defined personalization of Liuzijue therapeutics. Further integration of electrophysiological monitoring could elucidate neuromuscular coordination mechanisms during intervention, bridging traditional Qigong principles with contemporary neurorehabilitation science. We anticipate that more scholars will engage in foundational research in the field of Liuzijue and hope that this study can provide a reference for the development of the realm of science.

## 5 Conclusion

This study established an intervention approach suitable for animal research from the perspective of the primary kinematic characteristics of Liuzijue Qigong and evaluated its effects on lung function, respiratory muscle function, respiratory muscle structure, and the inflammatory, apoptotic, and protein synthesis profiles in respiratory muscles. Our results demonstrated that low‐intensity aerobic exercise, combined pursed‐lip breathing with aerobic exercise, and abdominal stimulation with aerobic exercise all significantly improved lung and respiratory muscle functions in a COPD mouse model. These beneficial effects are likely associated with the suppression of inflammatory responses and the modulation of the balance between muscle synthesis and degradation. The findings preliminarily support the use of composite stimulation as a rehabilitation intervention in the context of Liuzijue for COPD animal models. Despite the complexity of translating traditional Qigong to animal models, our stimulation paradigm successfully captured core therapeutic elements of Liuzijue. This paves the way for mechanistic studies on mind‐body interventions. Future research should prioritize exploring stimulus forms with higher fidelity for Liuzijue based on this framework, as well as investigating personalized Liuzijue protocols based on kinematic biomarkers for clinical translation.

## Author Contributions

**Jian Li:** methodology, writing – original draft. **Xiang Ji:** data curation, methodology. **Kangxia Li:** formal analysis, methodology. **Min Cao:** methodology. **Yuxin Sun:** software, writing – review and editing. **Chengbing Cao:** visualization, writing – review and editing. **Zifei Yin:** project administration. **Xin Wang:** resources. **Fanfu Fang:** resources, writing – review andediting. **Cai‐tao Chen:** conceptualization, funding acquisition, writing – review and editing. **Wei Gu:** conceptualization, writing – review and editing.

## Ethics Statement

The present study was approved by Science and Technology Ethics Committee of Tongji University (No. TJBH07824101). We confirmed that all experiments were performed in accordance with relevant guidelines and regulations and we ensured that manuscript reporting adhered to the ARRIVE guidelines (https://arriveguidelines.org) for the reporting of animal experiments.

## Conflicts of Interest

The authors declare no conflicts of interest.

## Supporting information


**Figure 1:** Results of COPD model verification.

## Data Availability

The datasets used and/or analyzed during the current study available from the corresponding author on reasonable request.
