# Supplementary figures and images for: An Animal Model of Liuzijue Based on Kinematic Features Exploration: A Pilot Study Conducting in COPD
Source: Immun Inflamm Dis. 2025 Aug 13;13(8):e70233. doi: 10.1002/iid3.70233 (PMC12344581; doi:10.1002/iid3.70233)

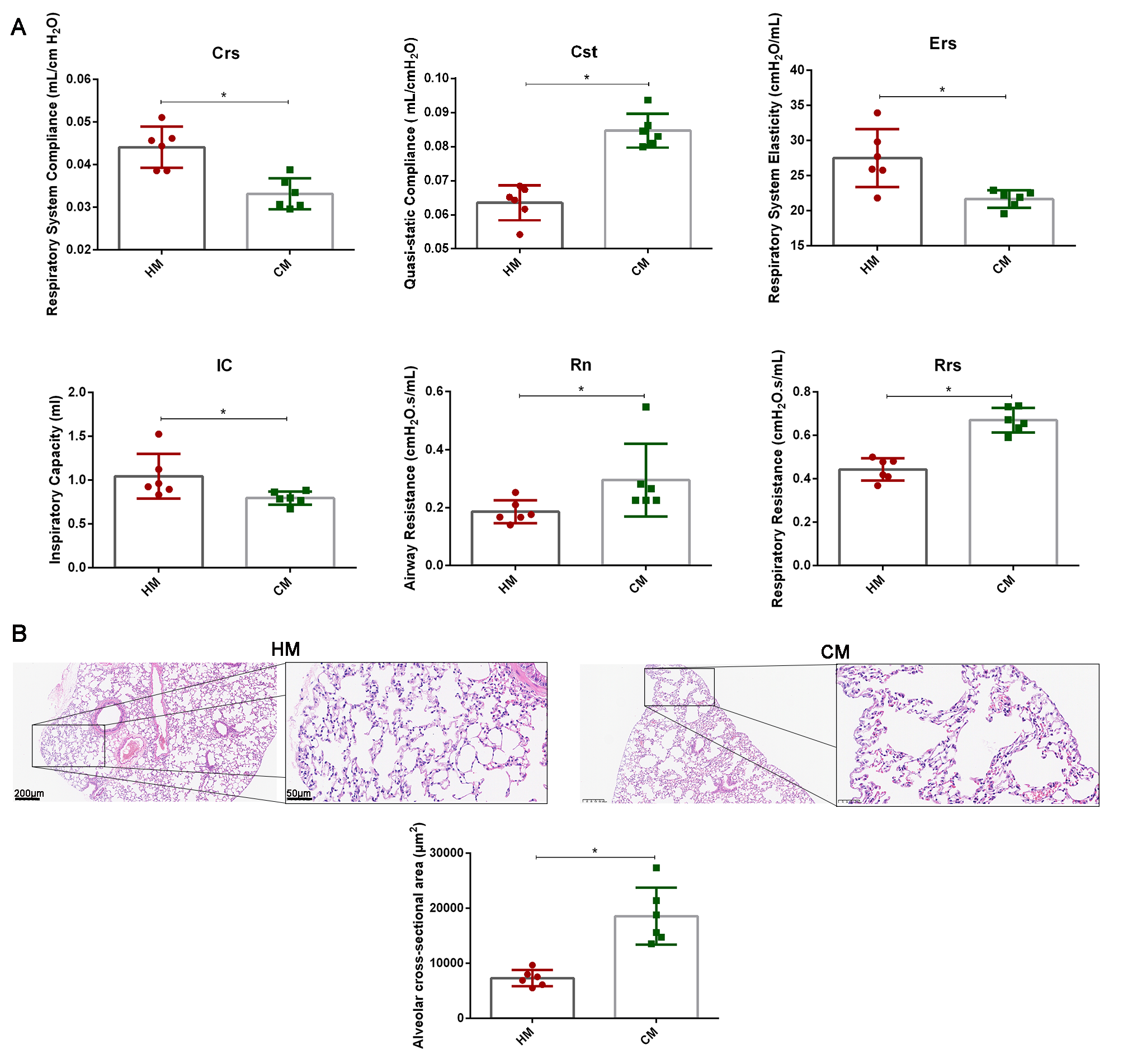

Supplement: Supplementary file 1 — Figure 1: Results of COPD model verification. [file IID3-13-e70233-s001.tif]
